# High Mobility Group A1 (HMGA1): Structure, Biological Function, and Therapeutic Potential

**DOI:** 10.7150/ijbs.72952

**Published:** 2022-07-04

**Authors:** Lu Wang, Ji Zhang, Min Xia, Chang Liu, Xuyu Zu, Jing Zhong

**Affiliations:** 1 Institute of Clinical Medicine, The First Affiliated Hospital, Hengyang Medical School, University of South China, Hengyang, Hunan, 421001, PR China.; 2 Cancer Research Institute, The First Affiliated Hospital, Hengyang Medical School, University of South China, Hengyang, Hunan, 421001, PR China.; 3 Department of Clinical Laboratory, Shenzhen Traditional Chinese Medicine Hospital, Shenzhen 518033, Guangdong, China.; 4 Department of Endocrinology and Metabolism, The First People's Hospital of Chenzhou, First School of Clinical Medicine, University of Southern Medical, Guangzhou 510515, Guangdong, China.

**Keywords:** High mobility group A1, Malignancies, Mechanisms, Chemoresistance, Targeting treatment

## Abstract

High mobility group A1 (HMGA1) is a nonhistone chromatin structural protein characterized by no transcriptional activity. It mainly plays a regulatory role by modifying the structure of DNA. A large number of studies have confirmed that HMGA1 regulates genes related to tumours in the reproductive system, digestive system, urinary system and haematopoietic system. HMGA1 is rare in adult cells and increases in highly proliferative cells such as embryos. After being stimulated by external factors, it will produce effects through the Wnt/β-catenin, PI3K/Akt, Hippo and MEK/ERK pathways. In addition, HMGA1 also affects the ageing, apoptosis, autophagy and chemotherapy resistance of cancer cells, which are linked to tumorigenesis. In this review, we summarize the mechanisms of HMGA1 in cancer progression and discuss the potential clinical application of targeted HMGA1 therapy, indicating that targeted HMGA1 is of great significance in the diagnosis and treatment of malignancy.

## Introduction

High mobility family A (HMGA) consists of three members: HMGA1, HMGA2 and HMGA3. HMGA1 is a nonhistone chromatin structural protein encoded by oncogenes and located in the nucleus. It can change the structure of DNA by binding to the rills of the A+T rich region in dsDNA and interact with transcription factors to regulate transcription. In addition to the expression of HMGA1 during normal development, HMGA1 is overexpressed in most malignancies, Furthermore, the malignant degree of tumours, chemotherapy resistance and drug sensitivity are related to the expression level of HMGA1.

## Overview of HMGA1

### Biological structure of HMGA1

High mobility group protein A1 (HMGA1) is called this because of its rapid migration in polyacrylamide gels. Due to the selective splicing of precursor mRNA, it can be divided into two subtypes: HMGA1a and HMGA1b, also known as HMGI/Y in previous literature 1. Human HMGA1a and HMGA1b are translated and encoded by the short arm of chromosome 6. The peptide chain length is approximately 100 amino acids (10.7-11.7kDa), belonging to low molecular weight histones. Structurally, it contains three independent conserved AT hook domains (DBDs) and an acidic carboxyl tail. The three positively charged AT hook domains are connected by linker peptides and can bind to AT-rich sequences in double helix DNA. Meanwhile, the P-R-G-R-P palindrome sequence of the AT hook ensures the specificity of the AT hook binding to DNA slots 2. The acid carboxyl terminal, including 30 consecutive Asp and Glu residues, negatively charged and regulates protein function mainly through phosphorylation.

The charge difference between the AT hook domain and an acidic carboxyl terminal has an effect on the conformation of DNA after binding to DNA, which can lead to DNA bending, straightening, unwinding, looping or topological structure. Then, through conformational changes, DNA can influence transcription by recruiting transcription factors into specific transcriptional regulatory regions through either enhancers or competition with histones or chaperones. In addition, compared with HMGA1a, HMGA1b lacks only 11 amino acid residues between the first and second AT hooks 3, and the rest of the sequences are roughly the same. Therefore, HMGA1a and HMGA1b are highly homologous in function, whether in the process of ageing, embryonic stem cells, type 2 diabetes mellitus or tumour development and progression will be involved in regulation. This regulation also relies on HMGA1 posttranslational modification such as phosphorylation, methylation, acetylation, ubiquitinylation and SUMOylation.

### Phosphorylation of HMGA1

HMGA1 is a structural building protein that regulates transcription by changing the conformation of DNA after binding with DNA. Moreover, posttranslational modification of the HMGA1 protein can change its binding affinity with DNA and regulate the function of the HMGA1 protein. Many studies have shown that HMGA1 is the substrate of most protein kinases and can be phosphorylated by tyrosine protein kinase (CK2), cdc2 kinase, protein kinase C(PKC), M-phase specific kinase (H1 kinase) and homeodomain-interacting protein kinase-2 (HIPK2) 4-8. For instance, the serine and threonine residues of HMGA1 are extensively modified, and the three serines (S98, S101 and S102), mainly located at the C-terminal acid carboxyl end, are phosphorylated by CK2 in a cell cycle-dependent manner 4, without affecting the acetylation and methylation of HMGA1 in vitro 9, 10. Moreover, the phosphorylation of HMGA1 at the S102 site can be a drug-resistant target in non-small cell lung cancer (NSCLC) resistant to tyrosine kinase inhibitor (TKI), and knockout of HMGA1 can transform TKI resistant NSCLC cells into TKI sensitive cells, which will be helpful for clinical treatment 11. The phosphorylation of HMGA1 protein by cdc2 kinase weakens the affinity of the N-terminal AT hook with promoter IFNbeta and the inherent bending state of IFNbeta 5. The phosphorylation of HMGA1 protein in purified maize by cdc2 kinase could also reduce the transcriptional inhibition mediated by histone H1, thus promoting the large-scale synthesis of zeins 12. HIPK2 can phosphorylate the ser-35, thr-52 and thr-77 of HMGA1a, thr-41 and thr-66 of HMGA1b and reduce the affinity with delta promoter; the degree of decrease is lower than for cdc2 as well. In addition, although the phosphorylation mediated by HIPK2 decreases the affinity of HMGA1a to DNA, the affinity increases in the absence of ATP 8. PKC alpha, beta, gamma and delta can phosphorylate HMGA1 and reduce its binding capacity with DNA fragments, thus changing its biological function 6. For example, in invasive and metastatic breast cancer, the phosphorylation level of HMGA1 is greater than that of low invasive primary cancer 13. Recent studies have shown that minichromosome maintenance protein 2 (MCM2), a factor related to the formation of a replication fork, can promote the proliferation of lung cancer cells by regulating the phosphorylation of ser-99 of HMGA1 14. At the same time, HMGA1, as a nuclear target downstream of the insulin receptor (IR) signaling pathway, can be phosphorylated to reduce transcriptional activation. When HMGA1 is phosphorylated, the expression of the insulin receptor gene is decreased, and it is more likely to develop type 2 diabetes 15. Diana et al. also reported for the first time that the phosphorylation of HMGA1a protein is related to apoptosis, where hyperphosphorylation occurs in the early stage and dephosphorylation occurs in the late stage with the formation of apoptotic bodies 16.

### Acetylation of HMGA1

Acetylation is also a common posttranslational modification of HMGA1, and acetylation does not affect CK2-catalysed phosphorylation 17. Using bottom-up proteomics, HMGA1 was hydrolysed into short peptides before mass spectrometry. The N-terminal lys-14 is acetylated 18. In addition, histone acetyltransferase P300 and P300/CBP associated factor (PCAF) could induce the acetylation of lys-14, lys-64, lys-66, lys-70 and lys-73 of HMGA1a, and the acetylation mode of HMGA1b was found to be similar 9. Acetylated HMGA1 is also a crucial structure for enhancer assembly. Enhancers can recruit histone acetyltransferase to activate transcription. HMGA1 can make the enhancer unstable or decompose through the acetylation of CBP at lys-65. The acetylation of PCAF in lys-71 can stabilize the enhancer and prevent the acetylation of CBP to enhance transcription. Two factors coordinate with each other to cause acetylation in HMGA1 to become a transcription switch 19. In addition, acetylation is detected in all AT hooks of the HMGA1a protein, but the peptide region between the second and third AT hooks is more easily acetylated than other regions. Moreover, HMGA1a isolated from metastatic breast cancer cells had a higher degree of acetylation than that isolated from nonmetastatic breast cancer cells 20. This also suggests that the modified HMGA1 protein is involved in the regulation of tumour progression 21.

### Methylation of HMGA1

HMGA1 is strongly methylated and upregulated in almost all cancer types. Epigenetic changes through GPG methylation of HMGA1 may regulate its gene expression in various cancers 22. It has been reported that arginine methylation of the HMGA1a protein may be linked to apoptosis, heterochromatin formation and tumour progression and be parallel to dephosphorylation in the process of apoptosis 18, 23, 24. PRMT1 and PRMT3 of the arginine methyltransferase (PRMT) family mainly methylates the arginine residue of the first AT hook of HMGA1a, and PRMT6 is a good substitute for HMGA1a methylation endogenous enzyme, mainly in the second AT hook methylation 10, 25. In addition, studies found that HMGA1a was monomethylated, symmetrically demethylated and asymmetric dimethylated in arg-25 26, and there were also monomethylation modifications independent of acetylation at lys64 and lys70 sites where acetylation can occur, while no methylation was found in HMGA1b 18. More importantly, methylation of HMGA1 arg-25 does not alter the total charge of residues, so it may not affect protein DNA binding considerably. In contrast, the introduction of hydrophobic methyl groups will affect hydrogen bonds and regulate the interaction between proteins 26.

### Ubiquitinylation and SUMOylation of HMGA1

**Fig 1:**
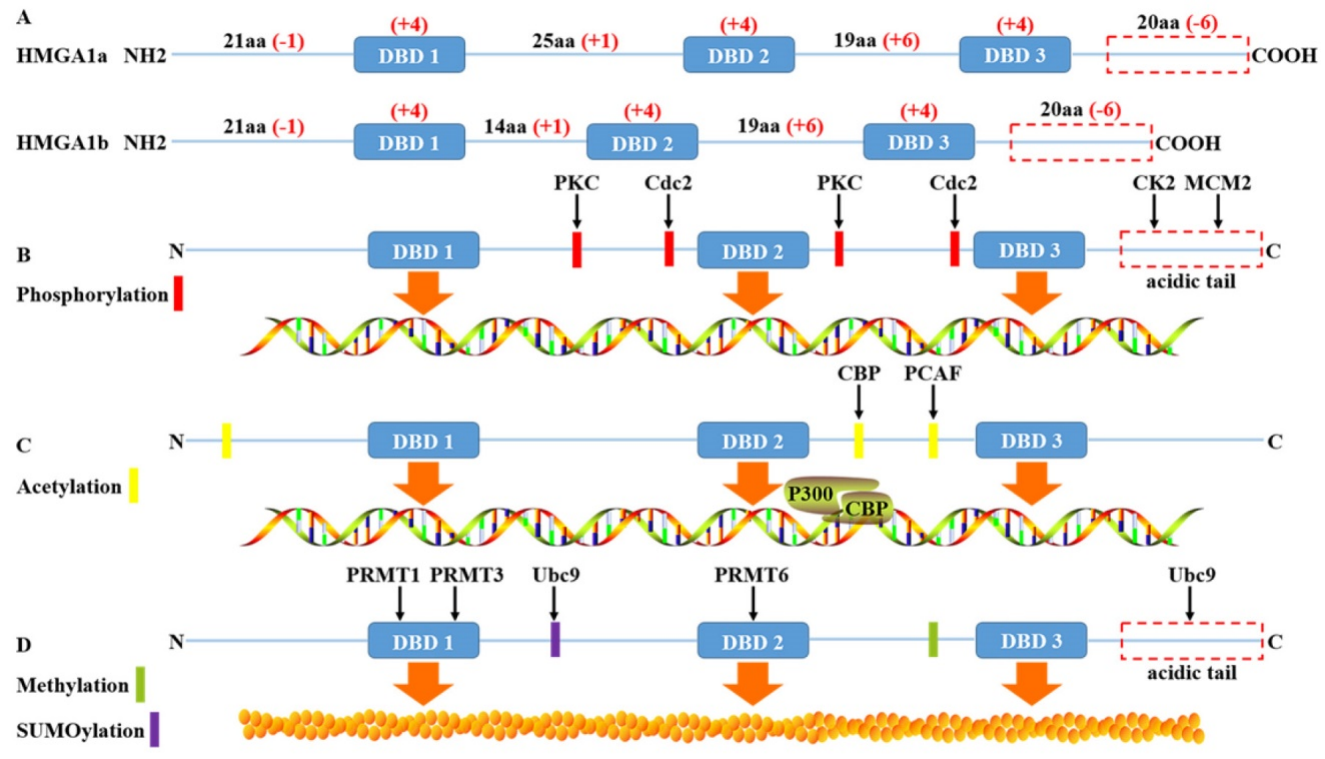
** The structure and post-translational modification of the HMGA1 protein.** (A) The human HMGA1 protein is divided into four functional domains: the DBD 1, DBD 2, DBD 3 and acidic C-terminal tail. The net charge distribution is shown in red. (B) HMGA1 protein is widely phosphorylated by the protein kinases PKC, Cdc2, CK2 and MCM2, affecting the affinity between HMGA1 and DNA to regulate HMGA1 function. The phosphorylation of HMGA1 by CK2 and MCM2 mainly occurs in the acidic C-terminal tail. (C) HMGA1 recruits the assembly of enhancer p300/CRP under the action of histone acetyltransferase CBP and PCAF to play the role of transcriptional regulation. (D) HMGA1 is methylated by PRMT family members and SUMO E2 Conjugase Ubc9 without being SUMOylated to regulate protein interactions.

Ubiquitinylation leads to proteasome-mediated degradation of the target protein, while SUMOylation more influences subcellular trafficking or the activity of target protein 27. GRP75 directly binds to HMGA1 to inhibit the ubiquitinylation and degradation of HMGA1, then upregulates HMGA1 to promote the proliferation and metastasis of lung adenocarcinoma cells by activating JNK/c-JUN signal 28. The proline-rich segment between AT hooks 1 and 2 and the C-terminal acidic domain of HMGA1b interacts with SUMO E2 Conjugase Ubc9 without being SUMOylated. Ubc9 binding only to the proline-rich segment of HMGA1b enhances the colony forming ability of rat1a cell, whereas the acidic domain negatively impacts on these properties 29. This also supports the view that HMGA1 has a dual role in some cases. In addition, HMGA1 also undergo ADP-ribosylation, often accompanied by DNA damage 30 (Fig. 1).

### Interaction between HMGA1 and histone

Histone H1 and HMGA1 are both nuclear proteins and contain specific DNA binding domains, where histones bind to DNA mainly through their own spherical domains 31, and HMGA1 regulates gene transcription by forming multiprotein complexes in promoter/enhancer regions or regulating chromatin structures 32. However, there is a certain antagonism between the two; HMGA1 increases the activity of DNA ligase IV by forming a complex with DNA-dependent protein kinase (DNA-PK) and counteracts the inhibitory effect of histone H1 on DNA ends, and overexpressed HMGA1 also causes rapid repair of broken DNA double strands 33. In addition, if cancer cells themselves wish to invade other tissues, they must be plastic. The nucleus is a hard organelle, and cancer cells will invade only when the structure changes. The nuclear building protein HMGA1 can phosphorylate the H1 histone, weaken the affinity between histones and DNA, relax chromatin, and finally achieve the purpose of changing the nuclear hardness and promote the invasion of cancer cells 34.

## The role of HMGA1 in cancer

### Breast cancer and reproductive system

#### Breast cancer

Breast cancer is the most common malignant tumour in women worldwide. Compared to other subtypes of breast cancer, triple negative breast cancer (TNBC) is the most aggressive and heterogeneous and often ends with organ-specific metastasis. It has certain resistance to conventional therapy, so prognostic markers and biotherapy are more helpful to the treatment of diseases 35. TNBC is negative for estrogen (ER), progesterone receptor (PR) and human epidermal growth factor receptor 2 (HER2) 36. There are likewise four subtypes in clinical practice: (1) luminal androgen receptor (LAR), (2) immunomodulatory, (3) basal-like immune-suppressed, and (4) mesenchymal-like 37. In breast cancer subtypes, HMGA1 has the highest expression in triple negative breast cancer, implying reduced overall survival 38, and its overexpression is positively correlated with HER-2/neu amplification and PR and negatively correlated with ER 39. In TNBC, Extracellular HMGA1 can be used as the ligand of receptor for advanced glycation end products (RAGE) to form an HMGA1-RAGE autocrine loop and induce pERK signalling to increase the migration and invasion ability of tumour cells 40. Cytoplasmic HMGA1 also inhibits p53-mitochondrial apoptosis and correlates with higher invasive tumor histotype 41. Moreover, when extracellular HMGA1 is blocked, cell adhesion will increase and migration ability will be weakened 42, which indicates that extracellular HMGA1 can be a drug target for TNBC and a biomarker for predicting distant metastasis. In addition, HMGA1a can regulate RAS-extractable signal-related kinase (RAS/ERK) signaling pathway related-genes, including KIT ligand (KL) and caveolins 1 (CAV1) and 2, and increase the sensitivity of MCF-7 cells to the RAS/ERK pathway to promote metastasis 43.

Studies have found that the HMGA1 gene does not rearrange and mutate in breast cancer. This indicates that overexpression is mediated by transcription activation or posttranscriptional regulation 44. Fra-1 can bind to the enhancer located in the last two introns of HMGA1 and upregulate the expression of HMGA1 mRNA at the transcriptional level, thereby enhancing the malignant metastasis of TNBC 45. TGF-β1 induces HMGA1 expression by activating HMGA1 promoter activity in MCF-7 and MDA-MB-231 cells, and specificity protein 1 (SP1) also participates in this process 46. FOXM1 stabilizes in the nucleus by forming a complex with HMGA1, which increases transcriptional activity to shared target genes. At the same time, they can cooperate to drive breast cancer cells to promote tumour angiogenesis to support invasiveness 47.

In addition, the imbalance of noncoding RNAs is a cause of the higher incidence rate and mortality rate of breast cancer, which can directly target HMGA1 to the progression of cancer 48. For example, let-7 is one of the earliest discovered microRNAs. Let-7a, a member of its family, can significantly downregulate the target gene HMGA1 to affect growth 49; miR-142-3p and miR-26a can be used as tumour suppressors to inhibit the expression of oncogene HMGA1 50, 51; miR-661 induces apoptosis after downregulating HMGA1 52; and miR-486-5p leads to G2/M cell cycle arrest and profound cell death of breast cancer cells by downregulating HMGA1 53. LINC00963 competes with miR-625 for endogenous RNA and attenuated the inhibitory effect of miR-625 on HMGA1 54. HMGA1 can also negatively regulate CBX7, and CBX7 negatively regulates the expression of miR-181b to affect the progression of breast cancer 55.

In breast cancer, the binding activity of HMGA1a and RNA can regulate the alternative splicing of estrogen receptor alpha (ERα) and reduce the sensitivity of tamoxifen-resistant cell lines to tamoxifen 56. HMGA1P6 and HMGA1P7, two pseudogenes of HMGA1 competing for endogenous RNA, can also lead to cancer when they are dysregulated, and the expression of HMGA1P7 is related to the levels of H19 and IGF2 in breast cancer, which is linked to the high double strand breaks in breast cancer cells 57. In addition, HMGA1 upregulates the expression of cyclin E2 (CCNE2) to alter Yap nuclear localization and downstream activity of the Hippo pathway and finally regulates the movement of basal-like breast cancer cells 58.

#### Ovarian cancer

Surgical resection and cisplatin chemotherapy are the preferred choices for the treatment of ovarian cancer, but because early screening factors are not obvious, symptoms only appear in the late stage, and the 5-year survival rate can be as low as 30% 59, 60. At present, studies have found that HMGA1 can regulate the expression of stem-related genes in ovarian cancer, such as KLF4, SOX2 and ABCG2, a drug-resistant protein, increasing the resistance of ovarian cancer to paclitaxel 61. HMGA1 can also regulate the expression of the KL promoter in ovarian cancer to maintain angiogenesis, which also indicates that serum KL levels can be used to help the diagnosis of HMGA1-positive carcinomas 62. HMGA1P6, as a competitive endogenous RNA of HMGA1, interferes with the effect of inhibiting microRNA in HMGA1 synthesis to enhance the malignancy of ovarian cancer cells, and MYC can participate in the transcription of HMGA1P6 63, 64. In contrast, let-7d-5p can negatively regulate the expression of HMGA1 and activate the p53 pathway, thereby inhibiting cell proliferation and promoting apoptosis and chemosensitivity 65. miR-638 negatively regulates HMGA1 to facilitate the cell cycle and suppress apoptosis 66. In addition, HMGA1 expression was not detected in epithelial cells, which are the common site of ovarian cancer, and compared with low malignant potential tumours, HMGA1 has a higher level in primary ovarian cancer, suggesting that HMGA1 has the potential to distinguish tumour biological behaviour 67.

#### Endometrial carcinoma

**Fig 2:**
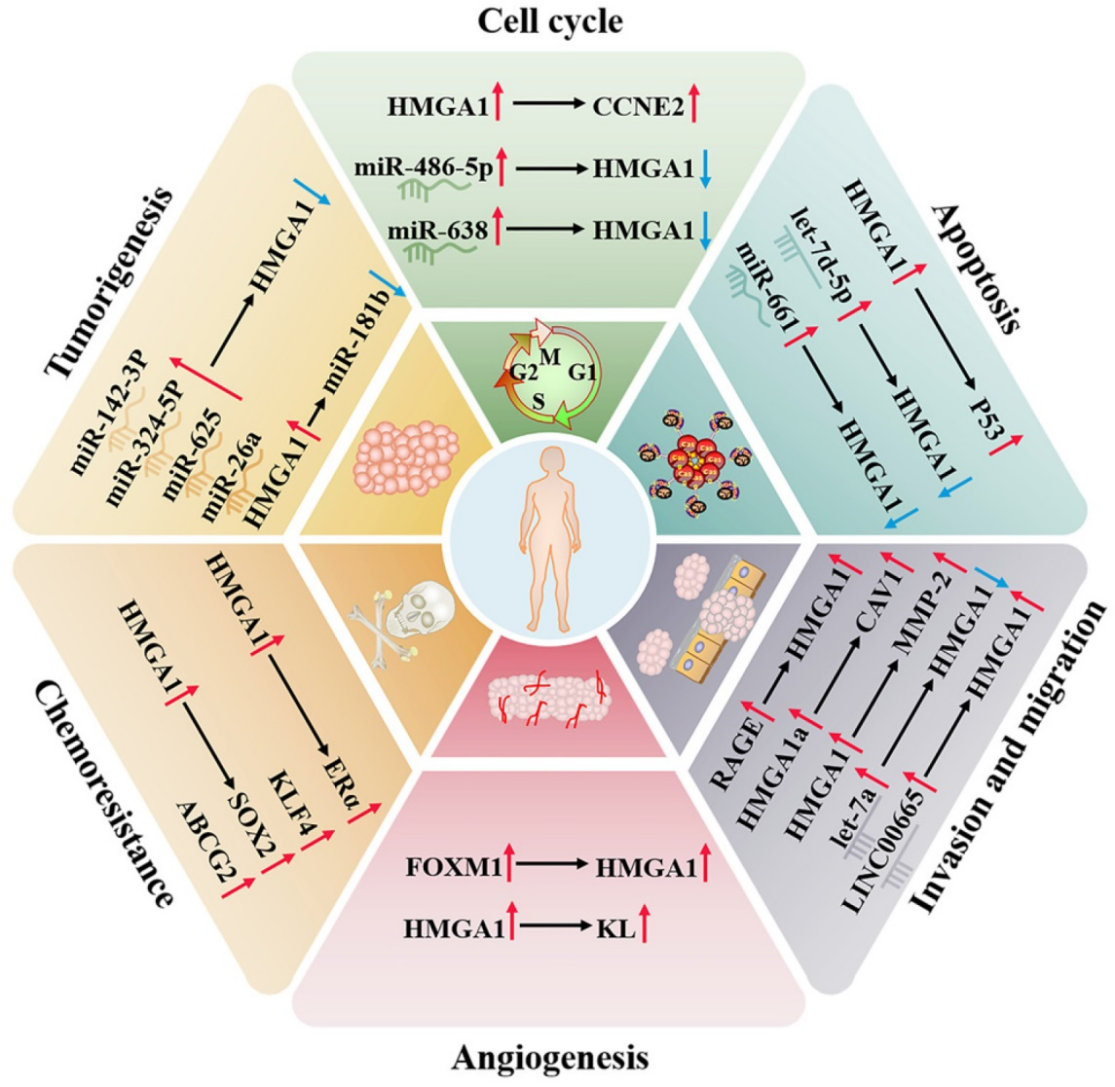
** Regulatory network of HMGA1 in the breast cancer and reproductive system.** HMGA1 can function as an oncogene, regulating the cell cycle, apoptosis, invasion, migration, angiogenesis, tumorigenesis and chemoresistance by interacting with ncRNAs and targeting genes and ultimately maintaining the malignancy of the reproductive system, making it a promising tumour biomarker.

Endometrial cancer is a common disease of the female reproductive system, which mostly occurs in the uterine epithelium of postmenopausal women. Cytology and transvaginal ultrasound are commonly used to detect endometrial cancer, but there is a lack of specificity 68, 69. The study confirmed that the protein and mRNA levels of HMGA1 are overexpressed in 46 cases of endometrial carcinoma (stages IA to IV), which is positively correlated with tumour stage, grade and size. The results were also verified in TCGA containing 381 endometrial carcinoma tumours (stages IA to IV), which implies that the overexpression of HMGA1 is a potential prognostic factor of endometrial carcinoma 70. What's more, the expression of HMGA1 pseudogenes increases with tumor stage progression, which is consistent with the expression pattern of HMGA1. It may be the result of the upregulation of HMGA1 pseudogenes suppressing the inhibitory effect of ceRNA on HMGA1 63, 70. HMGA1 is involved in nuclear translocation of β-catenin protein and is combined with MMP-2 promoter to participate in the invasion of endometrial cancer 71, 72. Both LINC00665 and circ 0067835 also upregulate HMGA1 to promote tumour progression. LINC00665 directly binds to the HMGA1 protein to promote cancer cell growth and invasion. Circ0067835 mainly induces the expression of HMGA1 by sponge miR-324-5p 73, 74. In addition, HMGA1 was truncated by the recurrent breakpoint of chromosome 12q15 translocation and then formed a fusion gene with ectopic DNA sequence to induce the occurrence of uterine leiomyoma 75 (Fig. 2).

#### Testicular germ cell tumors

Testicular germ cell tumors (TGCTs) are common malignant solid tumors in men aged 20 - 40 years and are one of the most common causes of death in solid tumors at this age 76. TGCTs are mainly divided into seminomas and nonseminomas germ-cell tumors (NSGCTs) 77. Previous studies have confirmed that HMGA1 has definite advantages in distinguishing the histologic groups of TGCTs. HMGA1 is only detected in seminomas and embryonic carcinoma belonging to NSGCTs, and HMGA1 is overexpressed in most cases, which is helpful in differential diagnosis 78. In addition, the overexpression of HMGA1 is partially regulated by the downregulation of Let-7a and miR-26a promoting proliferation, migration and invasion of seminoma cell line 79. ERβ is an important mediator of germ cell growth and development. The interaction between HMGA1 and androgen receptor co-regulator PATZ1 down regulates ERβ that puts TCam-2 cell line at a higher risk of malignant transformation 80. Moreover, after treatment with estrogens or estrogen-mimics, ERβ downregulation was also associated with increased cytoplasmic localization of HMGA1 81. In general, HMGA1 inhibitor will become a promising molecule to participate in the treatment of TGCTs.

### Digestive system

#### Liver cancer

HMGA1 as an oncogene, is often expressed in digestive tract tumours, such as liver cancer, colorectal cancer, pancreatic cancer and gastric cancer 82-85. In liver cancer, KIFC is located in the nucleus, and after being knocked down, the formation of internal foot and epithelial mesenchymal transformation can be reduced. TCF-4 is an important transcription factor in Wnt/β-catenin pathways, which enhances HMGA1 transcriptional activity by activating KIFC1, and then it promotes the pathogenesis of liver cancer and inhibits the sensitivity of liver cancer cells to paclitaxel 86. Other studies also confirmed HMGA1 increases monotonously from normal liver to liver cirrhosis and then to liver cancer, both in mRNA and protein levels 87. Overexpressed HMGA1 blocks the cell cycle and accelerates the proliferation and migration of cancer cells 88. Hepatocellular carcinoma with high expression of HMGA1 showed a significant increase in the amount of Macrophages M0, while M2 with repair activity decreased, indicating that HMGA1 can affect tumor immune infiltration and help tumor cells escape 89. Moreover, TRIM65, as an E3 ubiquitin ligase, can bind to Axin1 directly, ubiquitinate it and activate the β-catenin signalling pathway, while HMGA1, as an upstream factor of TRIM65, participates in the regulation 90.

#### Gastric cancer

In gastric cancer, HMGA1 and the c-Myc promoter contribute to induce c-Myc expression, thereby promoting aerobic glycolysis. After HMGA1 knockdown, glucose uptake and lactate production suffer as a result, and this change is only significant during glycolysis; there is no effect on oxidative phosphorylation in mitochondria, which suggests that HMGA1 regulation of gastric cancer metabolism has specificity 91. In addition, HMGA1 serves as a downstream target of Wnt/β-catenin pathways; after Wnt/β-catenin activates c-Myc, HMGA1 expression can be induced to maintain the activity of gastric cancer cells, which forms a positive feedback loop between HMGA1 and c-Myc 92. The downregulation of miR-195 combines with the HMGA1 3'- untranslated region (3' UTR) and induces its expression; this change in HMGA1 enhances the resistance of gastric cancer to 5-FU 93. In addition, lncRNA TRPM2 antisense RNA (TRPM2-AS) as a miR-195 molecular sponge also indirectly participates in the modulation of HMGA1 94.

#### Colorectal cancer

For colorectal cancer with a rising incidence rate, PD-L1 can evade immune surveillance by inhibiting the function of T cells. PD-L1 is also very expressed in colorectal cancer cells and interacts with HMGA1 to activate HMGA1-dependent pathways, mainly including the PI3K/Akt and MEK/ERK pathways, and it ultimately maintain the self-renewal of colorectal cancer cells 95. HMGA1 can also be induced by Ras, an oncogene in the distal regulatory region, which promotes the high expression of HMGA1 in tumour cells by activating the Ras GTPase signal and the cooperation between SP1 family members and AP1 factors 96. In addition, miR-214 is defined as a tumour suppressor due to its regulation of several abnormal signalling pathways. After poor expression in colorectal cancer, it can upregulate the activity of HMGA1 and its binding CTNNB1 by weakening the binding of the 3' untranslated region of wild-type HMGA1 to activate the Wnt pathway 97, 98. Sox9 is directly activated or indirectly upregulated by HMGA1 changing the chromatin structure to help construct the niche of Paneth cells, thereby amplifying the Wnt signal and maintaining self-renewal. Moreover, based on the overexpression of HMGA1, let-7 may be required for the development of Paneth cells 99.

In the study of faecal metabolomics of polyposis transgenic mice with abnormal expression of HMGA1, it was found that bile acid-related fatty acid metabolites in HMGA1 mice were significantly changed, and the synthesis of carnitine and Fas (myristic acid, palmitic acid, eicosenoic acid) increased, which helped the formation and maintenance of the cell membrane. Moreover, in some cancer samples, citric acid decreased, which reflects the possible existence of enhanced aerobic glycolysis 100. Similarly, due to the hypomethylation of CpG sites in some abnormal promoters, CAV1 overexpression in colorectal cancer is often associated with the enhancement of aerobic glycolysis. It can stimulate the transcription of glucose transporter SLC2A3/GLUT3 through the HMGA1 binding site in the promoter, increase glucose uptake and ATP production, and protect tumour cells from metabolic stress. At the same time, after consumption of CAV1, autophagy can be induced by activating AMPK-TP53/p53 101. These indicate the important role of HMGA1 in carcinogenesis by regulating metabolic pathways.

In addition, dysregulation of the cell cycle is a mechanism of tumorigenesis. The mitotic spindle assembly checkpoint (SAC) is an essential cell cycle control system that can help maintain genomic homeostasis in eukaryotic cells. HMGA1 activates the expression of the SAC gene, which also illustrates a new mechanism of HMGA1 overexpression leading to cancer 102.

#### Pancreatic cancer

Compared with normal acinar tissue or fibroinflammatory stroma, HMGA1 was significantly increased in pancreatic tissue with intraepithelial neoplasia 103. Periampullary carcinoma and pancreatic head carcinoma are two types of pancreatic-related cancers, but the expression of HMGA1 is higher in pancreatic head carcinoma and has no relationship with the later survival of patients 104. Meanwhile, HMGA1 is used as an independent predictor in predicting the survival of patients with pancreatic cancer; it induces tumorigenesis through the PI3-K/Akt mechanism and ensures resistance to gemcitabine and anoikis 83, 105, 106. When HMGA1 is silenced, there is a certain relationship between the activation of caspase 3 and the apoptosis induced by gemcitabine 105, and after HMGA1 overexpression, it can enhance the tumour invasion ability through Akt-dependent regulation of MMP-9 activity 107. COX-2 is able to convert arachidonic acid into prostaglandin, and HMGA1 combines with its promoter junction to upregulate its expression. It is worth noting that sulindac and celecoxib, which are COX-2 inhibitors, can significantly block the formation of pancreatic tumours 108. Metformin is a drug that can be used clinically to treat numerous diseases, such as type 2 diabetes 109, non-small cell lung cancer 110, and ovarian cancer 111. In the xenotransplantation model of nude mice, metformin can promote the expression of miR-26a, miR-192 and Let-7c in a dose-dependent manner, and upregulated miR-26a can inhibit the expression of HMGA1 and tumour growth, which also suggests why part of the biological effects of metformin play a role through HMGA1 112. In Ras signals that can regulate HMGA1, the downstream pathways of Ras and HMGA1 are different, which also explains that there is a synergistic effect between Ras and HMGA1 in transforming normal pancreatic epithelium. Among them, K-Ras mutation is mainly in the early stage of PanIN lesions, and HMGA1 occurs in the late stage, meaning that HMGA1 overexpression can promote the transformation of the premise lesion of K-Ras mutation into a tumour. Interestingly, neither HMGA1 nor K-Ras alone can transform pancreatic epithelial cells 108.

**Fig 3:**
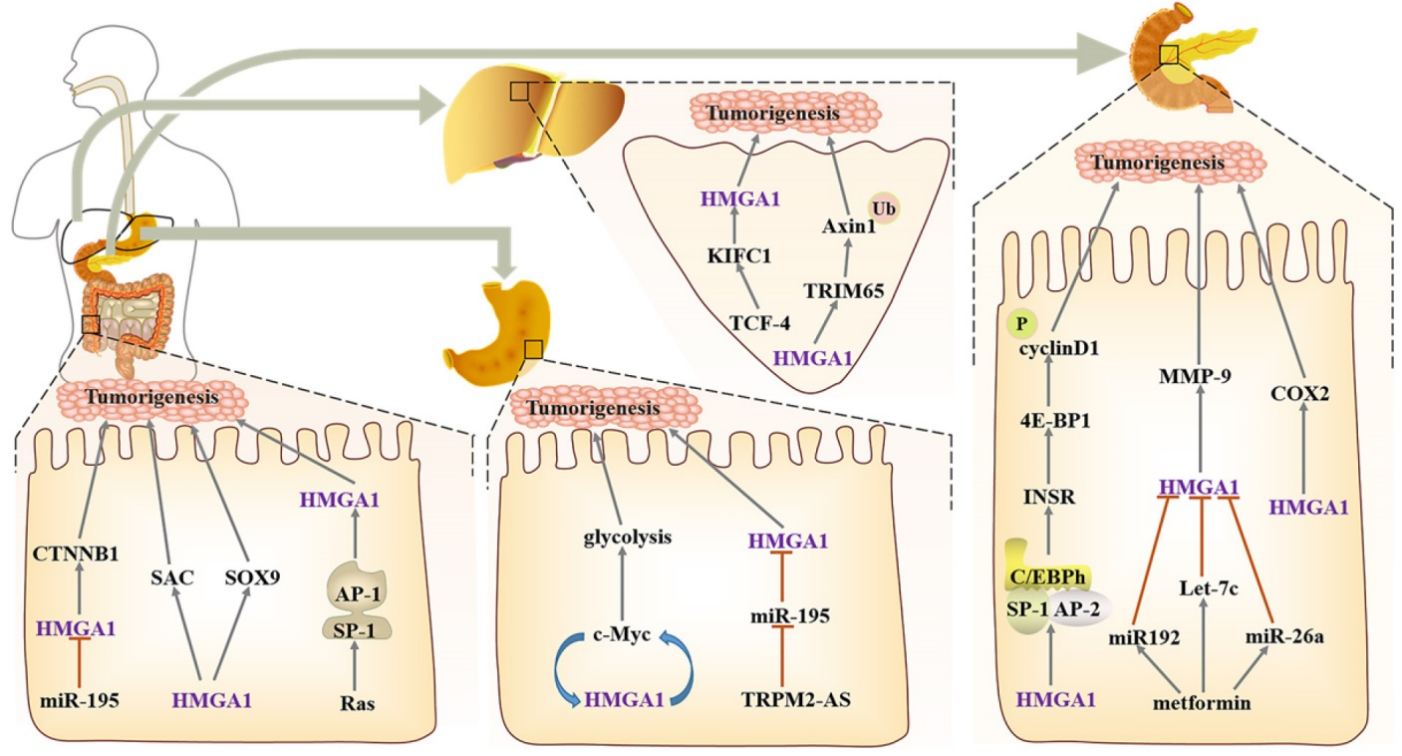
** Regulatory network of HMGA1 in the digestive system.** Liver cancer: TCF-4 enhances the transcriptional activity of HMGA1 and causes resistance to paclitaxel. HMGA1 acts on E3 ubiquitin ligase TRIM65 to activate the β-catenin pathway. Gastric cancer: HMGA1 and c-Myc form positive feedback in the regulation of glycolysis, and miR-195 negatively regulates HMGA1 to cause resistance to 5-FU. Colorectal cancer: HMGA1 upregulates the expression of CTNNB1, SAC and Sox9 to maintain the self-renewal of colon cancer cells. Pancreatic cancer, HMGA1 participates in the regulation of the cell cycle by interacting with the nucleoprotein complexes formed by SP1, AP2 and C/EBPh. Metformin can also indirectly participate in the regulation of HMGA1 to inhibit the progression of colorectal cancer. In addition, HMGA1 can increase the expression of COX-2 and promote pancreatic tumorigenesis.

One of the most important characteristics of pancreatic ductal carcinoma cells is the functional inactivation of the Rb-dependent G1 checkpoint. HMGA1 can accelerate the progression of the G1 phase and indirectly hasten the cell cycle of pancreatic ductal adenocarcinoma (PDAC). More importantly, HMGA1, SP1, AP2 and CAAT/enhancer binding protein h (C/EBPh) can form nuclear protein complexes to participate in the regulation of IR gene transcription factors, while IR can regulate the phosphorylation of 4E-BP1, thus controlling the translation of cyclin D1. Finally, the synergistic effect of cyclin D with CDK4/6 can also promote RB inactivation 113 (Fig. 3).

### Haemopoietic system

**Fig 4:**
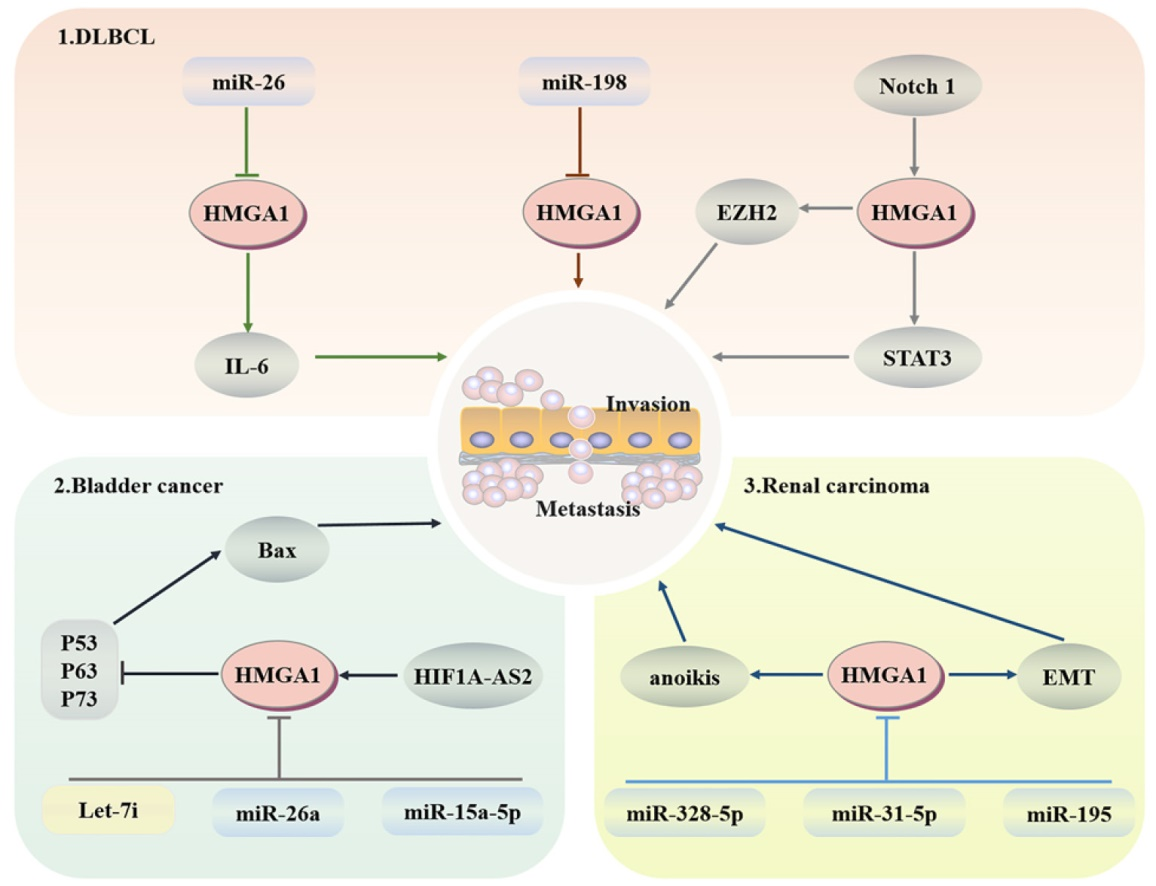
** Regulatory network of HMGA1 in the haematopoietic system and urinary system.** Haematopoietic system: HMGA1 mediates the imbalance of interleukin expression, resulting in B cell lymphoma. The Notch 1 signalling pathway associated with leukaemia activates HMGA1 to regulate cell proliferation. Bladder cancer: HIF1A-AS2 upregulates HMGA1 expression to inhibit apoptosis. MiR-26a targeting HMGA1 leads to cell cycle arrest. After HMAGA1 is inhibited by Let-7i and miR-15a-5p, the proliferation and migration of bladder cancer cells are also weakened. Renal cell carcinoma: HMGA1 promotes migration and metastasis by inducing anoikis and EMT.

Diffuse large B-cell lymphoma (DLBCL) is a common subtype of non-Hodgkin lymphoma that is invasive and is usually treated with the proteasome inhibitor botizomib. The potential mechanism is that bortezomib upregulates miR-198 in DLBCL, and then miR-198 combines with the 3'-UTR of AT-hook 1 to inhibit the expression of HMGA1, thus inhibiting the proliferation and migration of DLBCL cells 114. In addition, the imbalance of interleukin expression may lead to damage to T cell differentiation and expansion of B cell clones, resulting in B cell lymphoma. Enhancer of zeste-homolog 2 (EZH2), as an enzymatic subunit of PRC2, plays a cancer promoting role by catalyzing the methylation of histone H3 lysine 27 within PRC2 and regulating the G2/M transition of the cell cycle. HMGA1 combines with the promoter region of EZH2 to promote its transcription to enhance the proliferation and migration of B-cell lymphomas 115, 116. MiR-26 has been proven to be a multiple tumour suppressor; it does not control IL-6-related inflammation and tumour promoting activity through transcription inhibition but downregulates the production of IL-6 through HMGA1 and MALT1 silencing-induced NF-κB inhibition 117. HMGA1a targets STAT3 directly, whose activation can redisplay the transformation activity of HMGA1a to fibroblasts and promote the development of invasive lymphoid malignancy 118. After HMGA1 knockout, the transcriptional activity of the Rag2 protein of V-to-DJ involved in the IgH locus is increased, resulting in the formation of BCR complexes in mature B cells and an increase in the tumour susceptibility of lymphoid tissue 119. More importantly, the promoter region of HMGA1 can bind to the Notch1 signalling pathway related to leukaemia, and activated HMGA1 regulates cell proliferation through cell cycle regulation 120 (Fig. 4).

### Urinary system

#### Bladder cancer

LINC00649, which is located in the cytoplasm of bladder cancer, promotes the progression of bladder cancer by binding to miR-15a-5p to increase the expression of HMGA1 at the posttranscriptional level 121. HIF1A-AS2 upregulates the expression of HMGA1, thereby inhibiting BAX transcription through P63-, P73- and p53-dependent apoptosis 122. After HMGA1 silencing, downregulated p53 inducible nuclear protein 1 (TP53INP1) induces the activation of the ERK pathway in a p73-dependent DUSP10 manner. Moreover, TP53INP1 can also interact with LC3 to promote autophagic death, which is not linked to apoptosis 123. MiR-26a directly targets HMGA1 to block bladder cancer cells in the G1 phase, leading to cell cycle arrest and dyskinesia 124, 125. In addition, let-7i targets HMGA1 to significantly inhibit the proliferation and migration of T24 and 5637 bladder cancer cells, although let-7i is less expressed in bladder cancer 126.

#### Renal carcinoma

Studies have confirmed that HMGA1 is mainly expressed in the nucleus of renal carcinoma cells and induces anoikis. After HMGA1 overexpression, the colony-forming ability, invasion and migration ability of ACHN and Caki-1 renal carcinoma cells are enhanced 127. At the same time, HMGA1 can enhance the expression of miR-671-5p and induce epithelial mesenchymal transformation (EMT) by activating Wnt signalling 128. Furthermore, miR-328-5p, miR-31-5p and miR-195 have the capacity to prevent the progression of renal carcinoma by downgrading the expression levels of HMGA1 in the form of cell cycle stasis and EMT reversal 129-131. All the above studies indicate that HMGA1 may be a potential therapeutic target for renal carcinoma (Fig. 4).

#### Head and neck cancer

##### Thyroid cancer

Thyroid cancer is a common malignant endocrine tumour that is often diagnosed by biopsy in the clinic. However, studies have demonstrated that the molecular diagnosis of tumour-specific markers has higher accuracy and sensitivity 132. TGF-β1 can enhance the activity of the HMGA1 promoter and induce the expression of HMGA1 through extracellular signal-related kinase and phosphoinositide 3-kinase, so HMGA1 may exist as a tumour-specific marker for thyroid cancer 133. HMGA1 pseudogenes are difficult to detect in well differentiated thyroid cancer, and are only highly expressed in anaplastic thyroid carcinomas, and upregulate the level of HMGA1 protein to exert carcinogenic activity 134. S100A13, a calcium binding protein in the S100 family, regulates the proliferation and invasion of thyroid cancer by upregulating the expression of HMGA1 135. E2F1 interacts functionally with SP1 to promote HMGA1 expression. However, when SP1 binds to HMGA1 structurally, the balance between E2F family members is associated with the regulation of HMGA1 expression during quiescence and proliferation 136. In addition, HMGA proteins can also induce thyroid cancer transformation but have no impact on its phenotype. By speeding up the cells into S phase and delaying G(2)-M conversion, apoptosis is induced. Moreover, the HMGA1b acetylation site K60 and the third AT-hook are necessary regions to induce apoptosis 137. HAND1 in the Twist subfamily of Class B bHLH transcription factors is a key transcription factor that encodes trophoblast giant cell differentiation and heart tissue. Its promoter is methylated after binding with HMGA1, increasing the number of anaplastic cancer cell lines and promoting the progression of thyroid cancer 138. More importantly, the tumour suppressor p53 family is expressed in thyroid cancer, but the inhibitory activity in malignant tumours remains latent. HMGA1 is stably located in mitochondria and can resist the release of cytochrome c by replacing the binding of p53 and Bcl-2 and inhibit p53-mediated apoptosis in a transcription-independent manner 41. HMGA1 can be involved in the regulation of nuclear factor activity, depending on the interaction of the COOH-terminal oligomerization domain with all P53 family members, weakening the affinity between P53 and DNA, which also indicates that the tumour-promoting function of HMGA1 is partly dependent on the inhibition of tumour suppressor p53 activity 139.

##### Glioma

Glioma is a common malignant tumour of the brain. It originates from pluripotent neural stem cells and often develops into fatal brain cancer due to a large number of stem cells produced by symmetrical division. Numb has an asymmetric division of stem cells, and HMGA1 can negatively regulate the expression of Numb and promote the development of tumours by binding to the promoter of Numb or affecting miR-146a at the posttranscriptional level 140. Because HMGA1 is a structural transcription factor, it can change the expression of Sox2 through changes in the chromatin structure to drive the stemness of cells 141. Moreover, the stemness and temozolamide resistance of glioma cells also decreased after HMGA1 silencing 142. Zeng et al. indicated that the E-box element in the miR-637 promoter could target HMGA1 and vimentin to promote glioma after binding with zinc finger E-box-binding homeobox-2 (ZEB2) 143. As an inhibitor of glioma, miR-1297 can also target and negatively regulate HMGA1 to reduce cell viability 144.

##### Pituitary tumours

**Fig 5:**
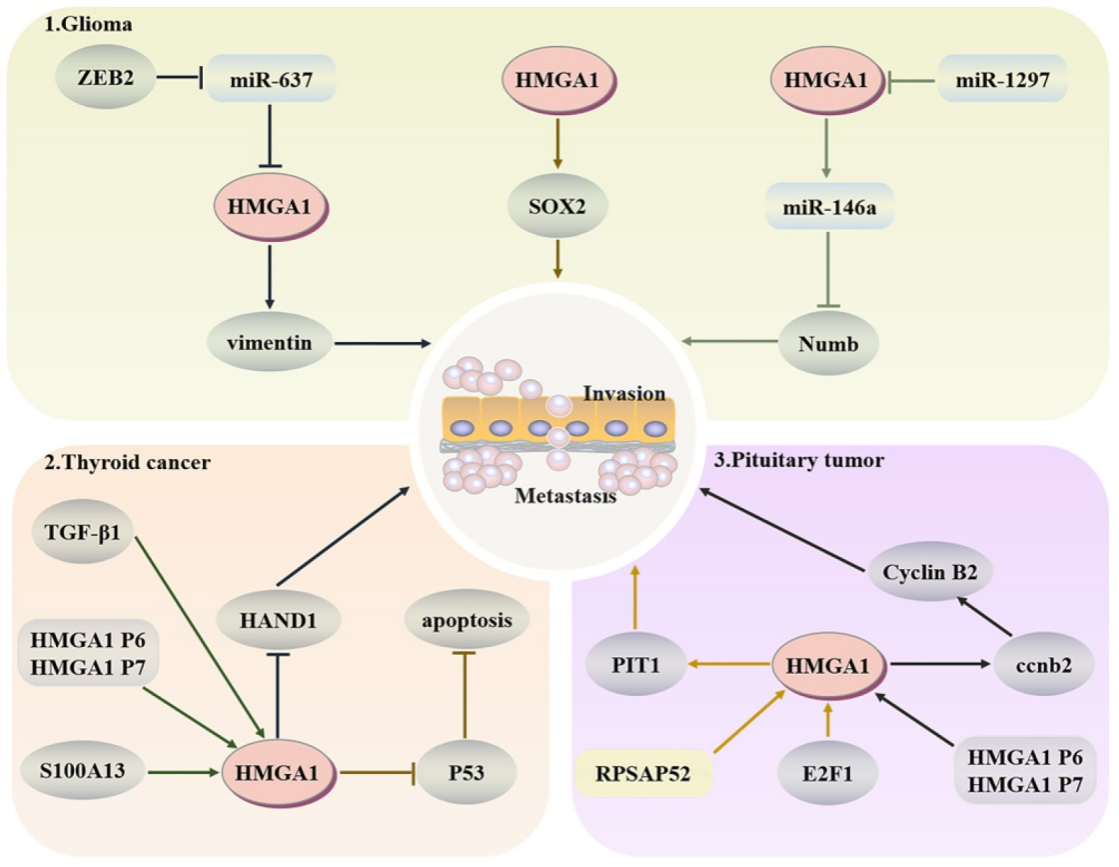
** Regulatory network of HMGA1 in the head and neck cancer.** Glioma: HMGA1 negatively regulates the expression of Numb to inhibit the asymmetric division of stem cells and positively regulates Sox2 to drive the dryness of cells. ZEB2 also targets HMGA1 and vimentin to promote glioma. Thyroid cancer: TGF-β1 and S100A13 upregulate the expression of HMGA1 to promote invasion and metastasis, and HMGA1 can inhibit p53-mediated apoptosis. Pituitary tumours: HMGA1 participates in the coding of CCNB2 to regulate the cell cycle. After E2F1 binds to HMGA1, PIT1, a key factor of pituitary development, shows a higher level in malignant tumours.

In pituitary tumours, HMGA1 can participate in the coding of CCNB2, produce more cyclin B2, and then regulate the cell cycle 145. RPSAP52 lncRNA can increase the protein level of HMGA1, accelerate the G1-S phase transition and promote tumorigenesis 146. PIT1, as a key transcription factor in pituitary development, after E2F1 binds to the HMGA1 promoter to increase the expression of HMGA1, and PIT1 tends to show a higher level in tumours 136. Esposito et al. confirmed that the HMGA1 pseudogene also upregulates the expression of HMGA1 in pituitary tumours to promote tumorigenesis 147. These results indicate that upregulation of HMGA1 will have a certain cancer-promoting effect in the pituitary (Fig. 5).

## HMGA1 and therapeutic strategies of HMGA1 involvement

### HMGA1 and Chemoresistance

Chemoresistance is a challenging issue in cancer therapy. Numerous studies have shown that HMGA1 can be involved in drug resistance to cisplatin, gefitinib, 5-FU, gemcitabine, and trabectedin in a TGF-β-, pathway-, microRNA-, and lncRNA-regulated manner in a variety of tumours, such as NSCLC, gastric cancer, ovarian cancer, glioblastoma, cholangiocarcinoma, colorectal cancer, thyroid cancer, and liposarcoma 65, 93, 142, 148-153. More importantly, HMGA1 silencing can also reduce self-renewal, dryness and spheroid formation in glioblastoma and colorectal cancer, increasing drug sensitivity 95, 142. As neo-adjuvant chemotherapy, trabectedin reduces HMGA1 expression in the treatment of myxoid LPS to play a potent anti-tumor activity. However, myxoid LPS resistant-cells upregulated the expression of HMGA1 and increased NFκB activity after treatment with trabectedin, resulting in tumor progression, but the cells will re-sensitive when drug-resistant cells treat with NF-κB, a NF-κB inhibitor 153. HMGA1 in pancreatic cancer is resistant to gemcitabine by an Akt-dependent mechanism, but stable short hairpin RNA targeting HMGA1 can resensitize drug-resistant cells 105. Therefore, blocking the expression of HMGA1 may be an effective target for chemotherapy resistance (Table 1).

### HMGA1 and Autophagy

Autophagy is the degradation of damaged organelles and misfolded proteins in lysosomes under pressure conditions to recycle the available components in cells to maintain cell homeostasis. Autophagy disorder is often shown in cancer and can inhibit or promote tumours 154. In most cases, cancer can be treated by cytotoxic autophagy, such as radiotherapy and chemotherapy, both of which enhance autophagy 155. Similarly, HMGA1 depletion increases autophagy and finally influences cancer cell viability by inhibiting the mTOR pathway and upregulating the transcription level of ULK1. However, there is no correlation between the number and maturity of autophagosomes, which leads to a decrease in autophagy flow and affects cell proliferation and survival 156. The ULk1 complex mainly induces autophagy by phosphorylating ATG13 and FIP200 157. In addition, the inhibition of HMGA1 can lead to the death of nerve cells partly through the decrease in autophagic flux induced by MPP^+^ 158. Downregulation of HMGA1 can also increase autophagy to inhibit proliferation and migration of bladder cancer by downregulating the level of miR-221 123. On the other hand, HMGA1 can directly increase the promoter activity of miR-222 to inhibit autophagy induced by the p27/mTOR pathway and finally aggravate the cardiomyopathy induced by high glucose 157.

### HMGA1 and Apoptosis Resistance

P53, a tumour suppressor gene, is mutated in most cancers. It mainly interacts with BCL-xL and Bcl-2 to change the permeability of the mitochondrial membrane to mediate apoptosis 159. HMGA1, as a nuclear structural factor, can transfer p53 proapoptotic activator homeodomain-interacting protein kinase 2 (HIPK2) from the nucleus to the cytoplasm, modulate the transcription of p53 target genes p21, Bax and Bcl-2, inhibit p53-mediated apoptosis, help cells escape apoptosis and produce cisplatin resistance 122, 160, 161. Moreover, the inhibitory effect of HMGA1 on p53-mediated mitochondrial apoptosis is mainly related to the inhibition of cytochrome C release and caspase activation. In other words, the cytoplasmic localization of HMGA1 in malignant tumours may be a new mechanism of p53 apoptosis inhibition 41. For example, HMGA1 knockdown by siRNA can control the growth and invasion of medulloblastoma by regulating apoptosis and forming pseudopodia and stress fibres 162. ShRNA-mediated HMGA1 silencing in BxPC3 activates caspase-3 and restores the chemosensitivity to gemcitabine 105.

Moreover, cell death caused by HMGA1 expression is mainly related to DNA repair damage. Therefore, Baldassarre and others also expressed that HMGA1 is highly expressed in malignant tumours, which helps malignant transformation from the perspective of DNA repair 163.

### HMGA1 and Senescence

In response to external pressure or cancer stimulation, cells stop replicating through the mechanism of ageing and inhibit tumour growth independently. Often, the activation of the tumour inhibitory pathway P53 or Rb, cell morphological changes, SA-β-galactosidase activity induction, enhanced protein secretion, and the formation of highly tight heterochromatin lesions are shown 164. HMGA proteins can change the chromatin structure through the AT hook domain and form senescence-associated heterochromatic foci (SAHFs) to maintain the stability of ageing 165, 166. Moreover, in mouse embryos overexpressing the HMGA1 P6 pseudogene, the mice grew faster and aged later, which was consistent with the results of pseudogene RNA crosstalk 167. Some studies have further shown that NOTCH can inhibit the formation of tight chromatin structures caused by HMGA1 and the accessible chromatin region driven by RIS autonomously or nonautonomously in ageing cells induced by Ras. At the same time, the chromatin structure of adjacent cells changes through the connection between cells 168.

### HMGA1 and Regulation of Stemness

Normal stem cells have the potential for multidirectional differentiation and proliferation. When their activity is hijacked by tumour cells, tumour cells can invade many parts of the body to escape treatment. Therefore, eliminating the stemness of cancer cells is another strategy for cancer treatment 169. HMGA1 protein is expressed at low levels or not expressed in adult tissues but is highly expressed in embryonic tissues with strong proliferation ability, which indicates that HMGA1 mainly plays a role in cells with strong proliferation ability 163. A large number of studies have shown that HMGA1 participates in the regulation of cancer diseases. For example, Wei et al. showed that PD-L1 upregulates the expression of HMGA1 and that the PI3K/Akt and MEK/ERK pathways related to HMGA1 were activated to favour the proliferation of colorectal cancer stem cells 95; Kim et al. confirmed that HMGA1 overexpression in ovarian cancer could increase the stemness of cancer cells, that cell proliferation, spheroid formation and gene expression related to stemness are enhanced, and that it also has higher resistance to doxorubicin and paclitaxel 61. Xian et al. indicated that HMGA1 can promote the expression of genes encoding Wnt receptors and Wnt downstream effectors and induce Paneth cells to differentiate into Sox9 to jointly establish an intestinal stem cell (ISC) niche 170. Wang et al. showed that the extracellular vesicles released by glioblastoma can cause neural stem cells (NSCs) to participate in the recurrence of glioblastoma again, and the imbalance of HMGA1 is involved in this process. All these enable us to find new methods for the treatment of this kind of disease 171. In addition, stem cells derived from human menstrual blood can regulate the abundance of 5-hydroxymethylcytosine (5-hmC) and 5-methylcytosine (5-mC) in enhancers and inhibit HMGA1 and other genes related to chemotherapy resistance 172. HMGA1 silencing reduces the stemness of glioblastoma and restores temozolomide sensitivity 142. HMGA1 itself can also promote the reprogramming of somatic cells to multifunctional stem cells, which will be useful for regenerative medicine and the treatment of poorly differentiated cancer 173.

### Targeting HMGA1 and RNA Crosstalk

Destruction of DNA is a basis for cancer treatment. However, due to the tolerance of cancer cells, most tumours will also relapse. HMGA1 plays a major role in tumours recurrence and is often associated with the prediction of poor prognosis 174. Clinically, doxorubicin, antiestrogen and other drugs target HMGA1 to achieve anticancer therapy 175, 176. Similarly, HMGA1 itself does not possess transcriptional activity, functioning primarily by changing the structure of the chromosome by binding the DNA region rich in the A-T hook. Dispatycin, a groove-binding drug, affects the binding of DNA rich in the AT region by changing the conformation and sequence specifically, thus alleviating the symptoms of inflammation during endotoxaemia 177. Adenovirus-mediated block of HMGA1 protein synthesis inhibited tumor growth, but had no effect on normal cells 178. In addition, many long noncoding RNAs, microRNAs and the two pseudogenes P6 and P7 of HMGA1 can interfere with the expression of HMGA1 by competing with the endogenous RNA of HMGA1. We can use them as target regulators to inhibit cancer development. For example, studies have confirmed that bortezomib upregulates the level of miR-198 to enhance the inhibition of HMGA1 by miR-198 to exert anticancer effects 114. Metformin upregulates miR-26a, which inhibits HMGA1 expression to affect pancreatic cancer progression 112. Epidrug mediates the re-expression of HMGA-targeting miRNA to intervene in the progression of pituitary adenomas 179. This further supports the strategy of targeted HMGA1 therapy.

### Small molecule inhibitor

Anti-gene therapy is another breakthrough in clinical drug resistance therapy. Polyetheylenimine polyplexes of spiegelmer NOX-A50 and minor-groove binder (MGB) netropsin compete for the binding of HMGA1 to AT-hook double-stranded DNA, thus affecting the regulation of HMGA 180, 181. Triplex-forming oligonucleotides (TFO1) and TFO-nitrogen mustard conjugates (TFO2) are two triplet oligonucleotides acting on the HMGA1 promoter. Although they are not stable with deoxynucleotides, they have a great regulatory effect on the transcription level of HMGA1. To improve the curative effect, it can be used together with adriamycin and other anticancer drugs, mainly because TFO is combined with the DNA groove, and adriamycin is embedded in the structure of DNA 182. The FR900482 class of antitumour drugs cross-links to the minor groove-binding HMGA1 protein to exert antitumour activity 183. However, these drugs partially affect the activity of HMGA1, so more specific HMGA1 inhibitors and targeted antibodies still need to be explored.

In addition, compared with the silencing of HMGA1 by shRNA and siRNA, the delivery of shRNA and siRNA by nanoparticles to block the transcription of HMGA1 does not interfere with AT binding protein in vivo 184.

## Conclusion and Prospects

HMGA1 has been confirmed to affect malignant tumours and has the potential to be a useful biomarker for the diagnosis and treatment of malignant tumours. In addition, HMGA1 is related to the chemoresistance of tumours. However, the function of HMGA1 still divided. For example, individual studies have confirmed that in MCF-7 cells with high HMGA1 expression, the sensitivity to chemotherapeutic drugs will be enhanced due to the decreased expression of BRCA1 with DNA repair activity, so the authors suggested that increasing the expression of HMGA1 can help the malignant transformation of BRCA1-expressing tumours 163. This may be based on the different cellular environment to affect the function of HMGA1 protein 185. In B-cell lymphoma caused by an imbalance in interleukin expression, knocking off the HMGA1 gene causes mature B cells to form the BCR complex, increasing the susceptibility of lymphoma 119. In fact, HMGA1 itself does not have transcriptional activity, which may be because its protein chaperones or regulated genes varies according to cell microenvironment and tumor heterogeneity. Therefore, the detailed role of HMGA1 needs further study.

HMGA1 is an important part of the progression of most tumours. HMGA1 can resist apoptosis and enhance senescence, which stops replication. HMGA1 may play a dual role in the process of disease development. In summary, because of the inconsistency of these functions, the mechanism by which HMGA1 causes these functions has yet to be explored to provide effective guidance for tumour diagnosis and targeted therapy.

## Figures and Tables

**Table 1:** Function of HMGA1 in different tumours

Disease	Interactors	HMGA1	Location	Pathway	Function	Reference
Lung adenocarcinoma Non-small cell lung cancer	GRP75	Up	nucleus	JNK/c-JUN	Proliferation and metastasis	28
	TGFβ1	Up	nucleus	-	Cisplatin resistance	148
	LINC01748	Up	nucleus	-	Increase aggressiveness	149
Gastric cancer	miR-195	Down	nucleus	-	Sensitive to 5-FU	93
	c-Myc	Up	nucleus	Wnt/β-catenin	Warburg effect	92
	TRPM2-AS	Up	nucleus		MiR-195 molecular sponge	94
Ovarian cancer	Let-7d-5p	Down	nucleus	p53	Sensitive to Cisplatin and apoptosis	65
	miR-638	Down	nucleus	-	Suppress apoptosis	66
Glioma	miR-637, miR-1297	Down	nucleus	-	Vimentin inhibition, reduce cell viability, sensitive to temozolomide	142-144
Pituitary tumours	RPSAP52	Up	nucleus	-	Accelerate the G1-S phase transition	146
	E2F1	Up	nucleus	RB/E2F1	Rb/E2F1 path imbalance	136
	HMGA1-pseudogene	Up	nucleus	-	Promote tumorigenesis	147
Cholangiocarcinoma	INOS, ERBB2	Up	nucleus	-	Gemcitabine resistance	150
	TMPO-AS1	Up	nucleus	-	Apoptosis resistance	151
Colorectal cancer	PD-L1	Up	nucleus	PI3K/Akt, MEK/ERK	Enhance dryness	95
	Ras	Up	nucleus	Ras GTPase	Activate Ras GTPase signal	96
	miR-214	Down	nucleus	-	Activate Wnt pathway	97, 98
	CAV1	Up	nucleus	Wnt	Warburg effect	101
Thyroid cancer	TGFβ1, S100A13	Up	nucleus	ERK	Proliferation and invasion	133, 135
	E2F1	Up	nucleus	RB/E2F1	Proliferation	136
Breast cancer	RAGE		extracellular	ERK	Migration and invasion	40
	Bcl-2		cytoplasm	-	Counteract apoptosis	41
	Let-7a, miR-142-3p, miR-625	Down	nucleus	-	Inhibition of proliferation and invasion	49, 51, 54
	miR-661	Down	nucleus	-	Apoptosis	52
	miR-486-5p	Down	nucleus	-	G2/M cell cycle arrest	53
Testicular germ cell tumors	Let-7a, miR-26a	Down	nucleus	-	Proliferation, migration and invasion	79
	ERβ	Up	cytoplasm	-	Malignant transformation	81
Liver cancer	TCF-4	Up	nucleus	-	Paclitaxel resistance, EMT	86
	TRIM65	Up	nucleus	β-catenin	Activate β-catenin pathway	90
Pancreatic cancer	miR-26a, miR-192, Let-7c	Down	nucleus	-	Inhibition of tumour growth	108
Endometrial carcinoma	LINC00665, circ 0067835	Up	nucleus	-	Cell proliferation, invasion and migration	73, 74
Diffuse large B-cell lymphoma	miR-198	Down	nucleus	-	Proliferation and migration	114
	miR-26	Down	nucleus	TNF-α/NF-κB	Control inflammation	117
Leukaemia	EZH2	Up	nucleus	-	Proliferation and migration	116
	Notch1	Up	nucleus	Notch1	Cell cycle	120
Bladder cancer	LINC00649	Up	nucleus	-	Accelerate progression	121
	miR-26a	Down	nucleus	-	Cell dyskinesia	125
	HIF1A-AS2	Up	nucleus	-	Suppress apoptosis	122
	Let-7i	Down	nucleus	-	Proliferation and migration	126
Renal carcinoma	miR-328-5p, miR-31-5p, miR-195	Down	nucleus	P-Akt, Wnt	Cell cycle stasis and EMT reversal	129-131

## Abbreviations

HMGA1: High mobility group A1
PI3K/Akt: Phosphatidylinositol 3 kinase/protein kinase B
MEK/ERK pathways: MAP kinase-ERK kinase/extracellular-signal-regulated kinase
ASP: Aspartic Acid
Glu: Glutamic acid
CK2: Tyrosine protein kinase
PKC: Protein kinase C
H1 kinase: M-phase specific kinase
HIPK2: Homeodomain-interacting protein kinase-2
NSCLC: Non-small cell lung cancer
TKI: Tyrosine kinase inhibitor
Ser: Serine
Thr: Threonine
MCM2: Minichromosome maintenance protein 2
IR: Insulin receptor
Lys: Lysine
PCAF: P300/CBP associated factor
CBP: Histone acetyltransferase binding protein
PRMT1: Arginine methyltransferase 1
PRMT2: Arginine methyltransferase 2
Arg: Arginine
DNA-PK: DNA-dependent protein kinase
TNBC: Triple negative breast cancer
ER: Estrogen receptor
PR: Progesterone receptor
HER2: Human epidermal growth factor receptor 2
LAR: Luminal androgen receptor
RAGE: Rceptor for advanced glycation end products
CAV1: Caveolin-1
RAS/ERK: RAS-extractable signal-related kinase
SP1: Specificity protein 1
ERα: Estrogen receptor alpha
IGF2: Insulin Like Growth Factor-2
CCNE2: Cyclin E2
Yap: Yes-associated protein
KLF4: Kruppel-like factor-4
SOX2: SRY-related high-mobility-group(HMG)-box protein-2
ABCG2: ATP binding cassette subfamily G member 2
KL: KIT ligand
MMP-2: Matrix metalloproteinase 2
TGCTs: Testicular germ cell tumors
NSGCTs: nonseminomas germ-cell tumors
KIFC1: Kinesin family member C1
TCF-4: T cell Factor 4
TRIM65: Tripartite motif‑containing 65
5-FU: 5-fluorouracil
TRPM2-AS: TRPM2 antisense RNA
PD-L1: Programmed death ligand 1
AP1: Activator protein 1
CTNNB1: Catenin beta 1
SOX9: SRY-Box Transcription Factor 9
SAC: Mitotic spindle assembly checkpoint
MMP-9: Matrix metalloproteinase 9
COX-2: Cyclooxygenase-2
PDAC: Pancreatic ductal adenocarcinoma
C/EBPh: CAAT/enhancer binding protein h
4E-BP1: 4E-binding protein 1
DLBCL: Diffuse large B-cell lymphoma
EZH2: Enhancer of zeste-homolog 2
PRC2: polycomb repressive complex 2
MALT1: Mucosa-associated lymphoid tissue lymphoma translocation protein 1
TP53INP1: p53 inducible nuclear protein 1
EMT: epithelial mesenchymal transformation
TGF-β1: transforming growth factor β1
E2F1: E2F transcription Factor 1
HAND1: Heart And Neural crest Derivatives expressed 1
ZEB2: Zinc finger E-box-binding homeobox-2
PIT1: Pituitary transcription Factor 1
ULK1: Unc51-like kinase-1
ATG13: Autophagy-related gene 13
MPP+: 1-Methyl-4-phenylpyridinium
HIPK2: Homeodomain-interacting protein kinase 2
SAHF: Senescence-associated heterochromatic foci
ISC: Intestinal stem cell
NSC: neural stem cell
5-hmC: 5-hydroxymethylcytosine
5-mC: 5-methylcytosine
MGB: minor-groove binder
TFO1: Triplex-forming oligonucleotide
TFO2: TFO-nitrogen mustard conjugate
BCR: B cell receptor
